# Using Coefficient to Distinguish Ambient/Focal Visual Attention During Cartographic Tasks

**DOI:** 10.16910/jemr.10.2.3

**Published:** 2017-04-03

**Authors:** Krzysztof Krejtz, Arzu Çöltekin, Andrew Duchowski, Anna Niedzielska

**Affiliations:** SWPS University of Social Sciences and Humanities, Warsaw, Poland; Department of Geography, University of Zürich, Switzerland; Clemson University, USA; National Information Processing Institute, Warsaw, Poland

**Keywords:** ambient/focal attention, coefficient *Κ*, cartography, route planning, visual search

## Abstract

We demonstrate the use of the ambient/focal coefficient *Κ* for studying the dynamics of visual behavior when performing cartographic tasks. Participants viewed a cartographic map and satellite image of Barcelona while performing a number of map-related tasks. Cartographic maps can be viewed as summary representations of reality, while satellite images are typically more veridical, and contain considerably more information. Our analysis of traditional eye movement metrics suggests that the satellite representation facilitates longer fixation durations, requiring greater scrutiny of the map. The cartographic map affords greater peripheral scanning, as evidenced by larger saccade amplitudes. Evaluation of *Κ* elucidates task dependence of ambient/focal attention dynamics when working with geographic visualizations: localization progresses from ambient to focal attention; route planning fluctuates in an ambient-focalambient pattern characteristic of the three stages of route end point localization, route following, and route confirmation.

## Introduction

Following construction of a map, via, e.g., selection, designation, classification, etc. (see Keates [29]), cartographers are interested in evaluating its use, including objective analysis of the user’s visual and/or cognitive engagement. Beyond measurement of a map’s intrinsic or visual complexity, which often relies on image-based measures related to saliency, clutter, or entropy (e.g., see Fairbairn [15]; Schnur, Bektas, Salahi, and Çöltekin [54]; Brychtová, Çöltekin, and Pászto [3]), it is important to find ways to measure perceived complexity, so that maps are well-suited to task types and to target user groups [56]. Št ˇ erba et al. suggest ˇ aiming psychological analyses to detect mechanisms and cognitive processes evoked during various tasks performed on maps, or cartographic products in general. Toward this end, we use K. Krejtz, Duchowski, Krejtz, Szarkowska, and Kopacz’s [37] *Κ* coefficient as a gaze metric to distinguish ambient and focal attention when performing cartographic tasks. In particular, our goal is to compare and contrast the use of a cartographically designed abstract map with its corresponding satellite image.

Due to the coupling between attention and saccades, saccade duration and amplitude are thought to reflect attentional selection and thus the spatial extent of parafoveal processing—peripheral scene degradation tends to curtail saccadic amplitudes [4]. Ambient attention is typically characterized by relatively short fixations followed by long saccades. Conversely, focal attention is described by long fixations followed by short saccades [59]. The *Κ* coefficient captures the temporal relation between standardized (z-score) fixation duration and subsequent saccade amplitude. *Κ* > 0 indicates focal viewing while *Κ* < 0 suggests ambient viewing. Fluctuating between focal and ambient modes, *Κ* could indicate changes in cognitive load corresponding to stimulus or task complexity, while, becoming more focal over time, *Κ* could indicate conclusion of visual search, and, for example, boredom, or the culmination of a decision.

In real-time applications, the *Κ* coefficient can potentially act as a contextual cue which could be exploited by software such as recommender systems, e.g., by not interrupting the user when in ambient search mode, or oscillating between ambient and focal search. Gaze-based recommender systems are designed to respond with information contingent on the viewer’s gaze, e.g., in geographic contexts, when directed to a particular location in physical or virtual space (such as on a map). Geographic gaze-based recommender systems have been referred to as location-aware (e.g., mobile) eye tracking systems [34]. For the system to provide an appropriate response, the system must identify the viewer’s desire for information through analysis of their gaze behavior. Generally, this is accomplished via computation of an interest metric [55, 46, 20]. Recent approaches characterize interest or boredom via Support Vector Machines [32] or Area Of Interest (AOI) revisitation [31].

We demonstrate the utility of *Κ* by comparing visual search behavior over two different geographic representations (a cartographic map and a satellite image) of the city of Barcelona alongside traditional eye movement metrics.

## Background

Among the many operations involved in map construction (see Keates [29]), an important aspect of map design is the control of the level of detail through generalization operations, e.g., via simplification [61]. Cartographers remove unwanted objects to deal with complexity, thus implicitly or explicitly acknowledge that visual clutter is undesirable, as it can negatively affect visual search [50, 63]. In general, the utility of a map depends on the amount of represented data: as visual density increases, so does information load, decreasing the map’s usability [56]

A map’s utility can in part be evaluated by considering visual search performance. Visual search is a fundamental function all sighted beings execute on a daily basis. We plan our paths at a glance to avoid danger or to find food and shelter. In other words, visual search is important for our survival. It is also a commonly tested task in the attention literature as visual search may be facilitated (or interrupted) by salient objects in our (central or peripheral) visual field [50]. Understanding human strategies in visual search is interesting to many professional groups such as psychologists, vision researchers, educators, designers, advertisers.

Visual search is also a fundamental (low-level) cartographic task [2]. Another term that cartographic literature uses for visual search is localization when a viewer is asked to find an object of interest on a map. This is a principal map use task, because regardless of the map type or the final goal of the map reader, an object or point of interest must be found before it can be studied further. Geographic task taxonomies widely acknowledge this task among the most basic and common [35, 5, 42].

The deployment of visual attention as well as its response to changing conditions is often linked to our cognitive state. In eye movement studies, overt visual attention is typically associated with the viewer’s point of gaze [18]. Since eye tracking devices can effectively capture only the central (foveal) gaze point, attempts to model visual behavior that is triggered by the global complexity of the visual stimuli, or signals received from peripheral vision, are studied only to a limited extent [41, 49].

Map complexity has been a topic of interest in cartography for many decades [14, 39, 16, 54]. However, there have only been a few attempts to study map complexity using eye movements. Castner and Eastman [6, 7] distinguished between focal and ambient processing (even though they did not use these terms), and emphasized the importance of this distinction in their studies. They utilized fixation duration as an indicator of “depth of cognitive processing” and interfixation distance as an indicator of “extent of peripheral processing”. They observed a correlation between what they termed imageability and perceived complexity, and concluded that eye movements are useful in assessing the “holistic properties of maps”.

Our work is conceptually similar to Castner and Eastman’s [7] in that we also distinguish between focal and ambient attention during cartographic tasks. However, while they used traditional eye movement metrics, e.g., derived from fixations (see Jacob and Karn [26]), we evaluate *Κ* for its applicability to analysis of cartographic tasks. Because task is known to influence eye movements [64], especially their dynamics [42], we test *Κ* under three different cartographic tasks, namely Localization, Point Of Interest, and Route Planning. These can be thought of as instances of Locate, Identify, and a combination of Associate and Correlate, respectively, using the cartographic task taxonomy found in Šterba et al. [56] (see also Knapp ˇ [35] and Wehrend and Lewis [60]).

Eye tracking experiments have investigated map-based wayfinding, suggesting that route planning (and route choice) are followed by a phase of transformation and encoding—see Kiefer, Giannopoulos, Raubal, and Duchowski [33] for a review. In this paper we consider route planning as one of a number of map-related tasks and demonstrate how *Κ* corresponds to different phases of route planning. Of six typical map-based visual tasks, namely free exploration, visual search, polygon comparison, line following, focused search, and route planning, Kiefer, Giannopoulos, Duchowski, and Raubal [30] found route planning and focused search to be the most cognitively demanding (as indicated by mean difference of pupil diameter with respect to the free exploration task, considered as baseline). Their work is possibly the most similar to our application of coefficient *Κ* to analysis of cartographic tasks.

## Attentional Dynamics

The *Κ* coefficient is derived by subtracting the standardized (z-score) fixation duration from the standardized amplitude of the subsequent saccade [37], reproduced here for convenience in Equation 1:

**Figure:**
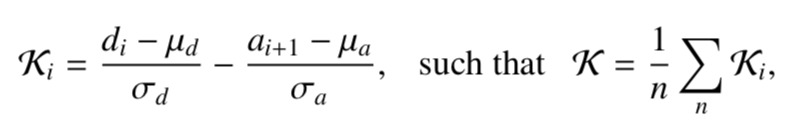


where µd, µa are the mean fixation duration and saccade amplitude, respectively, and σd, σa are the fixation duration and saccade amplitude standard deviations, respectively, computed over all n fixations and hence n Ki coefficients.

Similar combinations of fixation duration and saccadic amplitude have been proposed for the analysis of static and dynamic scene viewing [59, 36]. Specifically, short fixation durations combined with long saccades are characteristic of ambient processing, while longer fixation durations followed by shorter saccades are indicative of focal processing [58]. The pattern of visual attention attributed to the two ambient/focal modes of information acquisition [57] has been variably referred to as orienting and evaluating [24], noticing and examining [62], exploring and inspecting [59], skimming and scrutinizing [38], or exploring and exploiting [45].

The interplay between focal and ambient visual information processing changes dynamically. Shorter fixations followed by longer saccades appear to characterize early stages of scene perception. Once a target has been identified, longer fixations ensue and are followed by shorter saccades [25].

Using Velichkovsky et al.’s [59] terms of exploration and inspection, inspection may be comprised of decision and confirmation [28]. Pannasch, Helmert, Roth, Herbold, and Walter [44] showed a systematic increase in fixation durations and a decrease in saccadic amplitudes over the time course of scene perception. In their work, fixation durations and saccadic amplitudes were considered as two independent data streams. We combine both into a single dynamic stream explicitly capturing the interplay of ambient and focal modes of visual attention.

Holmqvist et al. [23] review several means of operationalization of ambient/focal viewing: thresholding on the ratio of fixation duration to saccade amplitude, and computation of a saccade/fixation ratio. None of these approaches, however, explicitly considers dynamics of how the saccade/- fixation ratio changes over time.

Our approach allows for both clear distinction of ambient (*Κ* < 0) and focal (*Κ* > 0) eye movements and its continuous dynamics. There is, however, an implicit ambiguity in *Κ* =0, which reflects effective equivalence between fixation duration and saccadic amplitude, relative to their z-scores, i.e., each is equivalent to its mean. The implicit ambiguity arises since *Κ* = 0 is neither focal nor ambient, however, its occurrence is rather rare (e.g., in the present study only 0.86% of the data fell within the *Κ* Ε [−0.01, 0.01] range).

In studying cartographic visual tasks, a measure of ambient/focal attention could indicate perceived complexity, task difficulty, or cognitive load. It is important to remember that various factors can contribute to understanding cartographic complexity, e.g., spatial abilities vary strongly among people [1, 21], expertise [8] and familiarity can change people’s strategies [19]. Furthermore, the map’s content and design also affects performance [9].

## Methodology

To evaluate effectiveness of a cartographic representation compared to its satellite rendering using coefficient *Κ*, we performed an experimental eye-tracking study of cartographic tasks. Three tasks were carried out by participants on a city view, displayed either as cartographic map or satellite rendering. Our hypotheses follow.

 1. The satellite view requires more attentive visual search, if it can be assumed to be more cognitively demanding. As such, we predict longer task completion times, longer fixation durations, and shorter saccades during inspection of this type of image compared to its cartographic representation. Moreover, we predict that the ambient/focal *Κ* coefficient will show more focal eye movements on the satellite image than on the cartographic map.

2. The route planning task elicits a different pattern of eye movement dynamics than the localization task. We assume the pattern of ambient/focal viewing reflects the different stages required to complete route planning: localization of the route start and end points (ambient viewing), traversing the route (focal viewing), and confirmation of the route (ambient viewing).

### Overview and Experimental Design

The experiment used a 3×2 mixed design, including cartographic task (Localization vs. Point Of Interest (POI) vs. Route Planning ) as a within-subjects factor and visualization (cartographic map vs. satellite rendering) as a betweensubjects factor. We also controlled for spatial working memory capacity (SWMC) of each individual (see below).

**Figure 1:**
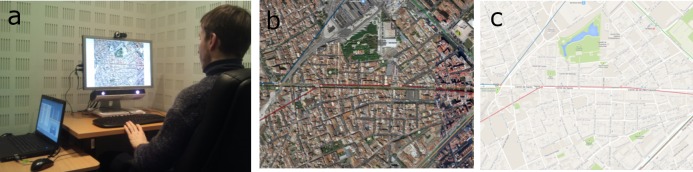
Experimental settings and (Barcelona) stimuli: satellite image and cartographic map. (a) Apparatus and experimental setting. (b) Satellite image. (c) Cartographic map.

The three cartographic tasks involved two types of visual search (Localization of a stated map landmark followed by search of a nearby POI) followed by Route Planning. Participants were asked to find locations on the map when viewing a city representation as either cartographic map or satellite image (Google’s cartographic or satellite rendering, respectively, see Figure 1 and below for technical details).

Note that for statistical analyses we skipped the POI task. The reason for this was the task’s simplicity. The task was to find a Point Of Interest close to the target of the first Localization task. Due to the POI’s proximity to the initial target, localization of the secondary POI was subsumed by the first task, making the distinction between stated hypotheses effectively meaningless.

### Participants

Sixty-three (N = 63) university students took part in the study, with 7 excluded due to technical and procedural problems (e.g., poor calibration). The final sample included 56 participants (20 M, 36 F, ages M = 25.43, SD = 3.94). Calibration scores for the sample were as follows: vertical M = 0.54◦ and horizontal M = 0.49◦. All participants took part in the experiment after signing a consent form.

### Apparatus

All stimuli were presented on a computer monitor (1680× 1050 resolution, 2200 LCD, 60 Hz refresh rate) connected to a standard PC laptop computer. Eye movements were recorded at 250 Hz with an SMI RED 250 eye tracking system. Stimuli presentation was controlled by SMI’s Experiment Centre software. SMI’s BeGaze software was used for fixation and saccade detection with a velocity-based event detection algorithm. The algorithm first detects saccades, then fixations. The minimum duration of saccades was set to 22 ms, with peak velocity threshold 40◦/s, and minimum fixation duration set to 50 ms. There is no consensus for fixation identification based on duration. For example, Velichkovsky et al. [59] consider a minimum fixation duration of 20 ms. However, other researchers consider fixations with larger minima, e.g., 100-200 ms [52, 43]. In the present paper, we applied a minimum fixation duration of 80 ms for the analyses (i.e., ignoring fixations with very short durations in range [50, 80] ms).

### Research Materials

Background questionnaire. A short online survey with the use of the LimeSurvey open-source platform [53] included questions about demographics, familiarity with Barcelona as well as Google Maps.

**Figure 2:**
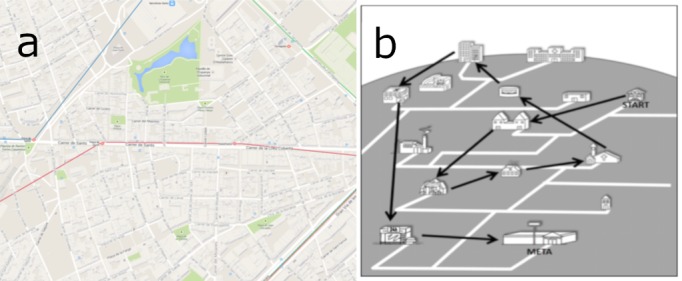
Examples of spatial working memory capacity tasks.(a) Spatial memory task 1. (b) Spatial memory task 2.

Spatial Working Memory Capacity. A Spatial Working Memory Capacity (SWMC) measure was adopted from the Berlin Test of Intelligence [27], following Dajlido [11]. We used two tasks for spatial working memory capacity measurement. Both were presented to participants in paper-and-pencil form. Example test boards are presented in Figure 2. We followed the test procedure and its timings provided by Dajlido [11].

In the first task, participants were asked to memorize, in 30 seconds, a path connecting 10 objects (buildings) presented on a board, see Figure 2(a). The buildings were shown on a background resembling streets. Afterwards, participants were presented with an answer sheet with only the buildings shown. Their task was to reproduce, with a pencil, the original path. The task was scored by a number of correctly reproduced connections in the path, resulting in the final score ranging from 0 to 10.

In the second task, participants were asked to remember the position of objects (buildings) presented on the test board, see Figure 2(b). The task involved memory of each object position as well as spatial relations between them. The time limit of this task was 45 seconds. During the test phase the task was to enter numbers assigned to each building in the proper empty spots on the answer sheet. All correct entries were summed for the final score ranging in [0, 12].

In order to obtain a final indicator of spatial working memory capacity, proportional scores from both tasks were averaged. They were then normalized to obtain a single score of spatial working memory capacity ranging between 0 and 1. The normalized score was used in subsequent statistical analyses as a covariant.

Experimental stimuli. The map stimuli (screen shots of the cartographic map and the satellite image) were created using Google Mapstm JavaScript API v3^1^, an Application Program Interface (API) made publicly (and commercially) available by Google, Inc. The API allows stylized rendering of a map through specification of JavaScript parameters. Maps were rendered (see Figure 1) by disabling visual user interface controls for navigation, scale, rotate, pan, and zoom, and limiting the number of Points Of Interest to two. Specifically, the Barcelona map (using Google’s latitude/longitude coordinates: 41.375384, 2.141004) displayed only the “park” and “sports_complex” POIs at zoom level 17 with the transit layer turned on. The maps were rendered to 1280×1024 resolution, then screen-captured and cropped to the same dimensions. The 1280×1024 images were fit vertically and centered on the 1680×1050 display, leaving grey margins on either side of the stimulus, see Figure 1(a). Centering the stimuli horizontally reduced the likelihood of eye movements made to distant horizontal screen locations, where eye tracking accuracy is lowest [40].

Note that because Google Maps manipulates which POIs are visible at discrete magnification (zoom) levels [10], it was impossible to control the selection of specific POIs at any given zoom level. Google Maps lacks application transparency and does not provide a means of determining which subset of existing POIs Google chooses to display. However, we controlled for this factor by fixing the zoom levels to a static number (17, in all cases).

### Procedure

Prior to the experiment, participants filled in an online background questionnaire on a laboratory computer. The tests for spatial memory were presented before or after the main experimental procedure to avoid order effects between their scores and the main part of the experiment.

In the main part of the experiment, participants were randomly assigned to either cartographic map (N=29) or satellite image (N = 25). Following this, the eye tracking system was calibrated to each individual. Participants were instructed to view a roving calibration dot which moved to successive screen coordinates covering the viewport extents. Following calibration, participants carried out the localization task (after having located the start point).

For Barcelona (see Figure 1), participants were given the scenario shown in Table 1. The first cartographic tasks were localization (visual search), the last included both localization and route planning. For brevity, we refer to the cartographic tasks as follows: Localization, and Route Plan-ning. To indicate selection of search targets, participants were asked to visually dwell on them for 3 seconds to indicate successful localization.

**Table 1:** Procedures for Barcelona stimulus

You have rented an apartment in Barcelona, located at the intersection of Carrer de Vilardell and Carrer d’Hostafrancs de Sió (first localization, or visual search task). Using either of the map or sat views, complete the following tasks:		
1.	[Localization.] Locate the apartment (street intersection) and fixate it for 3 seconds	
2.	[POI.] Locate the name of the closest metro station and fixate it for 3 seconds.	
You plan to go to the gym every morning at the Pavelló de l’Espanya Industrial sports complex (hint: large grey building with a domedroof abutting the large park of the same name). Using either of the map or sat views, complete the following tasks:		
3.	[Route Planning]. Plan a route that you’re likely to take to this complex from your apartment every morning:	
	a.	
	b.	Using the mouse, click on the apartment location.
	c.	Locate the sports complex, and, using the mouse, indicate the path you would take.
	d.	Using the mouse, click on the sports complex.
		Press the space bar when done.
		

Because some of the street names might have sounded foreign to participants (making it difficult to remember), a card with their names was made available by the display for reference. This was pointed out to participants just following calibration and prior to viewing of the stimulus. Participants were asked to view the stimulus (cartographic or satellite representation) as they would normally, and to balance speed and accuracy when performing the visual search.

All cartographic tasks were given in the same order as they were designed to follow a logical scenario. Both localization and route planning tasks were realistically achievable. In preserving their natural characteristics, cartographic tasks were self-paced and their completion time was not limited. This facilitated the use of the task completion time in analyses of performance.

### Dependent Measures

Results were analyzed in terms of task performance (effectiveness and efficiency) and eye movement characteristics and their dynamics. In particular, the following dependent variables were examined:

1. Task completion time (ms). We treated completion time (efficiency) as a main indicator of performance since all participants were able to complete all of the tasks successfully (effectiveness).

2. Fixation duration (ms). A classical measure in eyetracking research, averaged fixation duration is often treated as one of the indicators of cognitive resource management in visual information processing during scene viewing, e.g., see Henderson and Pierce [22], Rayner, Smith, Malcolm, and Henderson [48].

3. Saccade amplitude (deg). A classical measure of global vs. local visual information processing. Long saccades amplitudes are related to global visual scanning mode while short to a local search, e.g., Pannasch et al. [44], Unema et al. [58], Mills et al. [42].

4. Ambient/Focal *Κ* Coefficient. Derived by subtracting the standardized fixation duration from the standardized amplitude of the subsequent saccade, as expressed by (1), coefficient *Κ* was calculated for each participant. Negative values of *Κ* indicate relatively ambient viewing while positive values indicate relatively focal viewing. The higher the absolute value, the higher the ambient/focal magnitude [37].

For the statistical analyses of ambient/focal attention dynamics, task completion time was used as a within-subjects independent measure, where we divided each experimental task completion time into five equal periods for each participant. The five temporal periods were thus relative to the task duration, in other words, normalized with respect to task completion time, making them proportionately equivalent between tasks (see analysis of *Κ* below).

## Results

To verify hypotheses we used the Analysis of Covariance (ANCOVA) with spatial working memory capacity as a covariant, followed by pairwise comparisons with Tukey HSD correction when their effects reached statistical significance. All statistical computations were performed using the R statistical language [47].

### Familiarity with Barcelona and Google Maps

Familiarity with the city of Barcelona was evaluated with a question about the number of visits. The percentage of participants who had visited the city at least once in their lives was 24%. None of the participants indicated that they visited Barcelona more than 3 times in their life. One may conclude that overall familiarity with Barcelona was low.

Google maps was popular among participants, with only 7.1% claiming they had never used the service. Table 2 presents the detailed distribution of answers to the question “How often do you use Google Maps?” Observed performance (task duration) and process (visual attention) measures mainly represent attention and performance of experienced users of Google Maps. Our findings are thus most relevant when the location is new to the map user (e.g., as one would study a destination on a map prior to travel) but they are already familiar with the service.

**Table 2:** Responses to questionnaire question: “How often do you use Google Maps?” (N =56)

Response	Percent of responses
Every Day	7.4%
2-3 times a week	33.33%
once a week	12.96%
2-3 times a month	20.37%
one a month	18.52%
never	7.41%

### Cartographic Task Performance

All subjects successfully completed the tasks. To gauge task performance we studied task duration by analyzing basic eye movement metrics. We used a 2×2 ANCOVA with task duration as the dependent variable. The between-subjects predictor was the visualization (cartographic map vs. satellite rendering). The within-subjects predictor was the cartographic task (localization vs. route planning). Spatial working memory capacity was treated as a covariant.

As expected, analysis revealed a main effect of visualization type, F(1, 53) = 13.05, p < 0.001, η2 = 0.11. Both tasks took longer to complete with the satellite image (M=100842.03 ms, SE=598.92) than with the cartographic map (M=62342.85 ms, SE=466.57).

Analysis also showed a statistically significant main effect of covariant (spatial working memory capacity), F(1, 53) = 5.41, p < 0.05, η2 = 0.03. We performed a linear regression with task duration as a dependent variable and spatial working memory capacity as a predictor. Results showed that the slope of the regression line is significantly negative, β = −51328, SE = 21883, t(54) = 2.35, p < 0.05. Results imply, not surprisingly, that the higher the spatial working memory capacity, the faster the completion time for both localization and route planning tasks.

No other main or interaction effects were statistically significant (p>0.1).

### Fixation Duration

Task performance results indicated that the cartographic map afforded faster task completion. Analysis of process measures (i.e., eye movements) can help reveal whether task has an impact on performance. If route planning is the more cognitively demanding task, as suggested by Kiefer et al.’s [30] findings of increased cognitive load, then longer fixation durations would be expected during this task if, according to Just and Carpenter [28], they correspond to the duration of cognitive processing of fixated material. If the complexity of the visualizations has no impact, then similar task-dependent differences should be observed with both visualizations.

**Figure 3:**
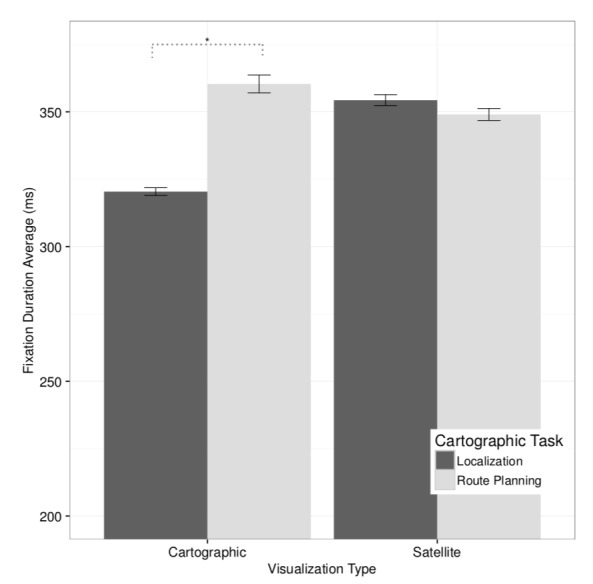
Interaction effect of task and visualization on average fixation duration. Whiskers represent ±1SE (standard error). Significant differences (p<0.05) are marked with ?.

To test these predictions we performed a 2 ×2 ANCOVA with average fixation duration as the dependent variable. The between-subjects predictor was the visualization (cartographic map vs. satellite image). The within-subjects predictor was the cartographic task (localization vs. route planning). Spatial working memory capacity was treated as a covariant. Analysis showed a statistically significant interaction effect between visualization (cartographic map vs. satellite image) and task (localization vs. route planning), F(1, 53) = 5.87, p < 0.02, η2 = 0.03, see Figure 3. Pairwise comparisons with visualization as moderator showed that, on the cartographic map, participants produced significantly longer fixations (M = 360.36 ms, SE = 3.30) while completing route planning than while performing the localization task (M=320.39 ms, SE=1.44), t(52)=2.24, p<0.03.

However, on the satellite image, the difference in average fixation duration was not statistically significant between the localization task (M = 354.32 ms, SE = 2.07) and the route planning task (M = 349.02 ms, SE = 2.22), t(52) = 1.16, p > 0.1. No other main or interaction effects were statistically significant.

### Saccade Amplitude

Because saccade amplitude and direction is thought to reflect attentional selection and the spatial extent of parafoveal processing (peripheral scene degradation tends to curtail saccadic amplitudes [4]), larger saccade amplitudes are expected in tasks that require greater parafoveal processing over stimuli that do not in some way degrade peripheral scene perception.

To evaluate the effect of cartographic visualization and task on saccade amplitude, a 2×2 ANCOVA was performed with saccade amplitude (in visual degrees) as the dependent measure. The between-subjects predictor was the visualization (cartographic map vs. satellite image). The withinsubjects predictor was cartographic task (localization vs. route planning). Spatial memory capacity was the covariant.

**Figure 4:**
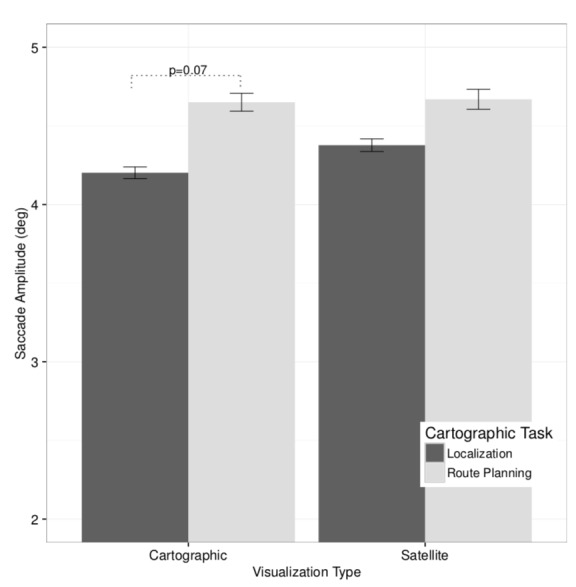
Marginally significant interaction effect of task and visualization on average saccade amplitude. Whiskers represent ±1SE (standard error).

Analysis revealed that the interaction of task and visualization was marginally significant, F(1, 53) = 3.38, p = 0.072, η2 =0.01, see Figure 4.

Pairwise comparisons with visualization as moderator showed that saccade amplitude is marginally greater during route planning (M = 4.65◦, SE = 0.06) than during localization (M = 4.20◦, SE = 0.04) on the cartographic map, t(52) = 1.78, p = 0.081. On the satellite image, the difference in saccade amplitude between the route planning task (M = 4.67, SE = 0.06) and the localization task (M = 4.38, SE = 0.04) was not statistically significant, t(53)=0.85, p>0.1.

It is worth mentioning that the interaction effect of task and spatial memory capacity reached marginal significance, F(1, 53)=3.48, p=0.068, η2 =0.01.

### Ambient/Focal Viewing

Preliminary analysis of the *Κ* ambient/focal coefficient as a dependent measure used a similar design as for fixation duration and saccade amplitude analyses, namely a 2×2 ANCOVA with visualization as a between-subjects factor and task as a within-subjects factor. Spatial working memory was treated as a covariant.

Analysis revealed a statistically significant main effect of visualization, F(1, 53) = 8.24, p< 0.01; η2 = 0.09, with the satellite image eliciting greater focal eye movements (M = 0.09, SE = 0.06) than the cartographic map, which elicited significantly greater ambient eye movements (M=−0.12, SE=0.04).

Similar to the analyses of fixation duration, the interaction effect of task and spatial working memory capacity reached marginal significance, F(1, 53)=3.53, p=0.066, η2 =0.02.

To delve deeper into the differences of dynamical patterns of ambient/focal fluctuation between localization and route planning tasks, two analyses of covariance were conducted, one for each of the cartographic and satellite visualizations. Both analyses followed the same 2×5 design with task and time sequence (5 periods) as within-subjects fixed factors. Spatial working memory capacity was treated as a covariant.

**Figure 5:**
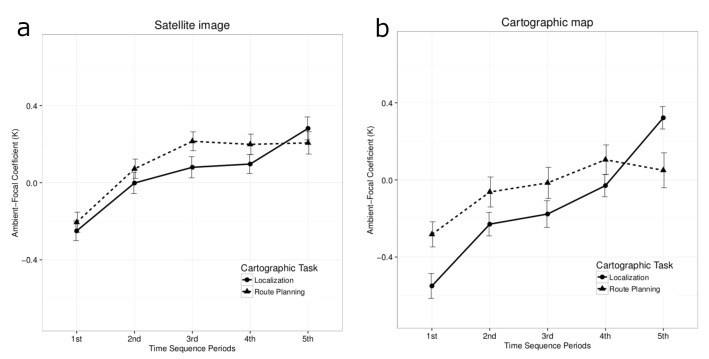
the user interface of ELAN. The software supports multiple synchronized media sources and an arbitrary number of annotation tiers. Videos are blurred to protect participants.(a) Coefficient *Κ* for task and time sequence on satellite image. (b) Coefficient *Κ* for task and time sequence on cartographic map.

Analyses of the satellite image revealed a significant main effect of time period, F(3.28, 81.94)=15.14, p<0.001, η2 = 0.13. In line with previous literature [59], pairwise comparisons showed that, regardless of task, attention changes from ambient to focal over time, see Figure 5(a): 1st period (M = −0.22, SE = 0.04), 2nd period (M = 0.03, SE = 0.04), 3rd period (M = 0.15, SE = 0.04), 4th period (M = 0.15, SE = 0.04), and 5th period (M = 0.25, SE=0.04). The difference between the 1st time period and all others was statistically significant, T1:T2 t(100) = 3.63, p< 0.01, T1:T3 t(100) = 5.23, p< 0.001, T1:T4 t(100)=5.90, p<0.001, and T1:T5 t(100)=7.33, p<0.001. The difference between T2 and T5 (t(100) = 3.69, p< 0.01) also reached significance.

Analysis of the cartographic map revealed similar effects, namely a significant main effect of time period, F(3.28, 88.55) = 14.00, p< 0.001, η^2^ = 0.13. Descriptive statistics also showed a similar progression from ambient to focal attention in the time course of both tasks, see Figure 5(b): 1st period (M = −0.44, SE = 0.05), 2nd period (M = −0.16, SE = 0.05), 3rd period (M = −0.11, SE = 0.06), 4th period (M = 0.02, SE = 0.05), and 5th period (M = 0.22, SE = 0.05). The difference between the first time period and the subsequent periods was statistically signifi cant, T1:T3 t(112.89) = 3.29, p< 0.01, T1:T4 t(112.89) = 5.70, p< 0.001, T1:T5 t(112.89) = 6.72, p< 0.001, T2:T4 t(112.89)=3.37, p<0.01, T2:T5 t(112.89)=4.39, p<0.001, and T3:T5 t(112.89) = 3.42, p< 0.01. Interestingly, a sig nificant interaction effect between task and time period was found, F(3.16, 85.25) = 5.24, p< 0.01, η^2^ = 0.07, see Fig ure 5(b). The following pairwise comparisons with time period as moderator showed that in the 2nd time period at tention is significantly more ambient during the localization task (M = −0.23, SE = 0.06) than during route planning (M = −0.06, SE = 0.08), t(125.11) = 2.04, p< 0.05. A similar marginally significant difference was found in the 3rd time period (M = −0.18, SE = 0.07 for localization and M = −0.02, SE = 0.08 for route planning), t(125.11) = 1.96, p=0.053. However, the pattern reverses in the last time period, with attention becoming significantly more ambient during route planning (M = 0.05, SE = 0.09) than during lo calization (M=0.32, SE=0.06), t(125,11)=3.01, p<0.01. 

Finally, the interaction effect of task and spatial working memory capacity reached significance for the cartographic map, F(1, 27) = 6.67, p< 0.01, η^2^ = 0.04. The following analyses of linear regression with coefficient *Κ* as the dependent variable and spatial working memory capacity as predictor for the localization task revealed a significant negative slope, β = −0.82, S E = 0.30, t(27) = 2.69, p< 0.02, while for route planning, the slope was positive but not significant, β = 0.36, S E = 0.36, t(27) = 0.99, p > 0.1, n.s:. Results suggest that higher spatial working memory capacity led to more ambient attention on the cartographic map but only during localization and not during route planning. Presumably, working memory serves as facilitator of visual search which dominates localization on a cartographic map. During route following perhaps more complex cognitive resources are involved beyond spatial working memory capacity. Further research is needed to investigate which cognitive resources are required for controlling the dynamics of visual attention during different tasks.

## General Discussion

Analysis of performance measures (efficiency and effectiveness, or speed and accuracy) show that the cartographic representation affords faster task completion than the satellite representation, regardless of task. Because everyone managed to complete all tasks, a ceiling effect precludes discussion of effect of task or cartographic product on accuracy. Of the two main tasks considered, route planning tended to be performed faster than localization, perhaps because both route endpoints had already been identified. The satellite image typically includes greater detail, and can be more cognitively demanding, than its cartographic counterpart. Results suggest a link between completion time and pattern of visual attention. Analysis of process measures (eye movements) provides further insights, yielding possible effects of cartographic product on cognitive requirements.

Analysis of fixation durations shows that task type has impact but only when using the cartographic map. This appears to agree with Mills et al.’s [42] observations of task influence. Results are also in line with the eye-mind assumption posited by Just and Carpenter [28], who pointed out that fixation duration corresponds to the duration of cognitive processing of fixated material. Salthouse and Ellis [51] also classically described a series of experiments showing that fixation duration is prolonged when participants are instructed to process visual information. The interaction effect of task and cartographic product on fixation duration suggests that the cognitive requirements of the satellite image override those of the task, i.e., the complexity of the satellite image obscures the effect of task.

Although route planning appeared to be performed faster (at a statistical tendency level), fixation durations show that route planning may have been more demanding than localization, as observed when using the cartographic map. This would be in agreement with Kiefer et al.’s [30] finding of increased cognitive load associated with route planning. Decreased saccade amplitude in route planning compared to localization, also suggests greater cognitive load, insofar as decreased amplitude suggests greater focal viewing, as also indicated by *Κ*.

Extending traditional fixation duration metrics, coefficient *Κ* fosters understanding of the dynamics of eye movements as revealed in differing patterns between both tasks. The localization task produced a fairly common dynamical pattern, with eye movements initially ambient, becoming more focal over time. When close to locating the target on the map, eye movements become more focal with the ratio between saccade amplitude and fixation duration leaning towards the latter. The route planning task, however, yielded a more complex dynamical pattern, but only over the cartographic map. Starting in ambient mode when locating the start and end points of the route, eye movements become focal when following the route visually, then finally turn more ambient during route confirmation. The use of *Κ* showed the three stages of route planning: route end point localization, route following, and route confirmation. However, the complexity of the satellite image again obscures this progression.

Just and Carpenter [28] noted that eye fixation data make it possible to distinguish the three stages of visual search performance, although their analysis relied on the relation between fixation duration and angular disparity. While qualitatively effective, the relation provided no easy way of combining fixation duration and disparity into a useful quantity with which to distinguish the cognitive stages. The difference in cartographic product notwithstanding, our *Κ* metric [37] illustrates when these inter-stage transitions may occur. When *Κ* < 0, relatively short fixations are followed by relatively long saccades, suggesting ambient processing during visual search. When *Κ* > 0, relatively long fixations are followed by short saccade amplitudes, suggesting focal processing during decision-making. Subsequent gaze transitions may indicate confirmation, as noted by Just and Carpenter [28].

## Conclusions

We presented a demonstration of how traditional gaze metrics can be augmented by analysis of dynamic attention with coefficient *Κ* to study differences in cartographic tasks while examining the utility of cartographic maps versus their satellite image counterparts.

We showed how traditional gaze metrics of fixation durations and saccade amplitudes help explain differences in performance observed during cartographic tasks. Specifically, we observed performance and gaze behavior differences among participants as they worked with satellite and cartographic representations. Performance results regarding the cartographic map suggest a nuanced outcome corroborating earlier work, suggesting impoverished performance using satellite images [12, 17, 13].

The benefits of cartographic maps were explained to a certain extent by fixation durations and saccadic amplitudes. On average, fixations were shorter on cartographic maps than on satellite images, likely facilitating faster cognitive processing, assuming Just and Carpenter’s [28] eye-mind assumption. Fixation durations and saccade amplitudes were also able to indicate task differences, suggesting route planning as the more demanding task due to the significantly longer fixation durations and (marginally) larger saccade amplitudes employed compared to the localization (search) task.

Beyond traditional eye movement metrics, which describe visual behavior over the duration of the task (in the aggregate or mean), coefficient *Κ* showed how the tasks differed over the course of their execution. The localization task elicited a fairly common dynamical pattern with gaze initially ambient, becoming more focal over time. The route planning task on the cartographic map, however, yielded a more complex pattern potentially resembling Just and Carpenter’s [28] search ! decide ! confirm progression.

## Acknowledgements

We would like to thank Mr. Janusz Arabski, undergraduate student of the University of Social Sciences and Humanities in Warsaw, Poland for his help in conducting the study.

The authors declare that there is no conflict of interest regarding the publication of this paper. 

## Footnotes

https://developers.google.com/maps/ documentation/javascript/, last accessed May, 2015. Google Maps provides an alternative API for producing static images, however, this API (v2) lacks the richness of features provided by the JavaScript API, which is why the latter was chosen.

## References

[b1] Allen G. L. (1999). Spatial Abilities, Cognitive Maps, and Wayfinding: Bases for Individual Differences in Spatial Cognition and Behavior In Golledge R. G. (Ed.), Wayfinding Behavior: Cognitive Mapping and Other Spatial Processes (pp. 46–80). The Johns Hopkins University Press.

[b2] Boér A., Çöltekin A., & Clarke K. C. (2013). An Evaluation of Web-based Geovisualizations for Different Levels of Abstraction and Realism—What do users predict? In Proceedings of the International Cartographic Conference (pp. 209–220). Dresden, Germany.

[b3] Brychtová A., Çöltekin A., & Pászto V. (2016). Do the visual complexity algorithms match the generalization process in geographical displays? ISPRSInternational Archives of the Photogrammetry, Remote Sensing and Spatial Information Sciences, 375–378. Retrieved from http://dx.doi.org/10.5194/isprs-archives-XLI-B2-375-2016 doi:

[b4] Cajar A., Schneeweiß P., Engbert R., & Laubrock J. (2016). Coupling of attention and saccades when viewing scenes with central and peripheral degradation. Journal of Vision (Charlottesville, Va.), 16(2), 8. 10.1167/16.2.8 1534-7362 27271524

[b5] Carter J. R. (2005). The many dimensions of map use. In Proceedings of the International Cartographic Conference.

[b6] Castner H. W., & Eastman R. J. (1984). Eye-Movement Parameters and Perceived Map Complexity–I. American Cartographer, 11(2), 107–117. 10.1559/152304084783914768 0094-1689

[b7] Castner H. W., & Eastman R. J. (1985). Eye-Movement Parameters and Perceived Map Complexity–II. American Cartographer, 12(1), 29–40. 10.1559/152304085783914712 0094-1689

[b8] Çöltekin A., Fabrikant S. I., & Lacayo M. (2010). Exploring the efficiency of users’ visual analytics strategies based on sequence analysis of eye movement recordings. International Journal of Geographical Information Science, 24(10), 1559–1575. 10.1080/13658816.2010.511718 10.1080/13658816.2010.511718 1365-8816

[b9] Çöltekin A., Heil B., Garlandini S., & Fabrikant S. I. (2009). Evaluating the Effectiveness of Interactive Map Interface Designs: A Case Study Integrating Usability Metrics with Eye-Movement Analysis. Cartography and Geographic Information Science, 36(1), 5–17. 10.1559/152304009787340197 1523-0406

[b10] Dähn A., & Cap C. (2014). Application Transparency: How and Why are Providers Manipulating Our Information? IEEE Computer, 47(2), 56–61. 10.1109/MC.2013.187

[b11] Dajlido P. (2013). Przetwarzanie Materiału Realnego lub Abstrakcyjnego: Konstrukcja Testów w Ramach Nowego Wymiaru Pomiaru Kompetencji Poznawczych (Unpublished master’s thesis). University of Social Sciences and Humanities, Warsaw, Poland.

[b12] Dillemuth J. (2005). Map Design Evaluation for Mobile Display. Cartography and Geographic Information Science, 32(4), 285–301. 10.1559/152304005775194773 1523-0406

[b13] Dong W., Liao H., Roth R. E., & Wang S. (2014, February). Eye Tracking to Explore the Potential of Enhanced Imagery Basemaps in Web Mapping. The Cartographic Journal, 1743277413Y.000. doi: 10.1179/1743277413Y.0000000071

[b14] Eastman J. R. (1977). Map Complexity: An Information Approach (Unpublished doctoral dissertation). Queen’s University, Kingston, ON, Canada.

[b15] Fairbairn D. (2006a). Measuring Map Complexity. The Cartographic Journal, 43(3), 224–238. Retrieved from http://dx.doi.org/10.1179/000870406X169883 doi:

[b16] Fairbairn D. (2006b). Measuring Map Complexity. The Cartographic Journal, 43(3), 224–238. 10.1179/000870406X169883 0008-7041

[b17] Francelet R. (2014). Realism and Individual Differences in Route-Learning (Unpublished master’s thesis). University of Zürich.

[b18] Goldberg J. H., & Kotval X. P. (1999). Computer Interface Evaluation Using Eye Movements: Methods and Constructs. International Journal of Industrial Ergonomics, 24(6), 631–645. 10.1016/S0169-8141(98)00068-7 0169-8141

[b19] Golledge R. G., Dougherty V., & Bell S. (1995). Acquiring Spatial Knowledge: Survey Versus Route-Based Knowledge in Unfamiliar Environments. Annals of the Association of American Geographers, 85(1), 134–158. 10.1080/00045602409356894 0004-5608

[b20] Hammer J. H., Maurus M., & Beyerer J. (2013). Realtime 3D Gaze Analysis in Mobile Applications In Proceedings of the 2013 conference on eye tracking south africa (pp. 75–78). New York, NY: ACM; Retrieved from http://doi.acm.org/10.1145/2509315.2509333, 10.1145/2509315.2509333

[b21] Hegarty M., & Waller D. A. (2005). Individual Differences in Spatial Abilities In Shah P. & Miyake A. (Eds.), The cambridge handbook of visuopatial thinking (pp. 121–169). Cambridge University Press 10.1017/CBO9780511610448.005

[b22] Henderson J. M., & Pierce G. L. (2008). Eye movements during scene viewing: Evidence for mixed control of fixation durations. Psychonomic Bulletin &Review, 15(3), 566–573. Retrieved from http://dx.doi.org/10.3758/PBR.15.3.566 doi:18567256

[b23] Holmqvist K., Nyström M., Andersson R., Dewhurst R., Jarodzka H., & Van de Weijer J. (2011). Eye Tracking: A Comprehensive Guide to Methods and Measures. Oxford University Press.

[b24] Ingle D. (1967). Two visual mechanisms underlying the behavior of fish. Psychologische Forschung, 31(1), 44–51. 10.1007/BF00422385 0033-3026 5605116

[b25] Irwin D. E., & Zelinsky G. J. (2002). Eye movements and scene perception: Memory for things observed. Perception & Psychophysics, 64(6), 882–895. 10.3758/BF03196793 0031-5117 12269296

[b26] Jacob R. J. K., & Karn K. S. (2003). Eye Tracking in Human-Computer Interaction and Usability Research: Ready to Deliver the Promises In Hyönä J., Radach R., & Deubel H. (Eds.), The Mind’s Eye: Cognitive and Applied Aspects of Eye Movement Research (pp. 573–605). Amsterdam, The Netherlands: Elsevier Science 10.1016/B978-044451020-4/50031-1

[b27] Jäger A. O., Süß H. M., & Beauducel A. (1997). Berlin test of intelligence. Göttingen: Hogrefe.

[b28] Just M. A., & Carpenter P. A. (1976). Eye Fixations and Cognitive Processes. Cognitive Psychology, 8(4), 441–480. 10.1016/0010-0285(76)90015-3 0010-0285

[b29] Keates J. S. (1982). Understanding maps. Burnt Mill. Harlow, Essex, UK: Longman Group Limited.

[b30] Kiefer P., Giannopoulos I., Duchowski A., & Raubal M. (2016). Measuring Cognitive Load for Map Tasks through Pupil Diameter. In Proceedings of the Ninth International Conference on Geographic Information Science (GIScience 2016). Springer International Publishing. 10.1007/978-3-319-45738-3_21

[b31] Kiefer P., Giannopoulos I., Kremer D., Schlieder C., & Raubal M. (2014). Starting to get bored: An outdoor eye tracking study of tourists exploring a city panorama In Proceedings of the 2014 Symposium on Eye Tracking Research and Applications (pp. 315–318). New York, NY: ACM; Retrieved from http://doi.acm.org/10.1145/2578153.2578216, 10.1145/2578153.2578216

[b32] Kiefer P., Giannopoulos I., & Raubal M. (2013). Using eye movements to recognize activities on cartographic maps In Proceedings of the 21st ACM SIGSPATIAL International Conference on Advances in Geographic Information Systems (pp. 488–491). New York, NY: ACM; Retrieved from http://doi.acm.org/10.1145/2525314.2525467, 10.1145/2525314.2525467

[b33] Kiefer P., Giannopoulos I., Raubal M., & Duchowski A. T. (2017). Eye Tracking for Spatial Research: Cognition, Computation, Challenges. Spatial Cognition and Computation, 17(1–2), 1–19. 10.1080/13875868.2016.1254634 1387-5868

[b34] Kiefer P., Straub F., & Raubal M. (2012). Towards location-aware mobile eye tracking In Proceedings of the 2012 Symposium on Eye Tracking Research and Applications (pp. 313–316). New York, NY: ACM; Retrieved from http://doi.acm.org/10.1145/2168556.2168624, 10.1145/2168556.2168624

[b35] Knapp L. (1995). A Task Analysis Approach to the Visualization of Geographic Data In Nygeres T. L., Mark D. M., Laurini R., & Egenhofer M. J. (Eds.), Cognitive aspects of human computer interaction For geographic information systems (pp. 355–371). Kluwer Academic Publishers 10.1007/978-94-011-0103-5_25

[b36] Krejtz I., Szarkowska A., Krejtz K., Walczak K., & Duchowski A. T. (2012, March 28-30). Audio Description as an Aural Guide of Children’s Visual Attention: Evidence from an Eye-Tracking Study. In Proceedings of the 2012 Symposium on Eye Tracking Research and Applications. New York, NY: ACM 10.1145/2168556.2168572

[b37] Krejtz K., Duchowski A., Krejtz I., Szarkowska A., & Kopacz A. (2016). Discerning Ambient/Focal Attention with Coefficient K. Transactions on Applied Perception, 13(3).

[b38] Lohmeyer Q., & Meboldt M. (2015). How We Understand Engineering Drawings: An Eye Tracking Study Investigating Skimming and Scrutinizing Sequences. In Proceedings of the International Conference on Engineering Design. Milan, Italy.

[b39] MacEachren A. M. (1982). Map Complexity: Comparison and Measurement. American Cartographer, 9(1), 31–46. 10.1559/152304082783948286 0094-1689

[b40] Mantiuk R. (2017). Accuracy of High-End and Self-build Eye-Tracking Systems In Kobayashi S., Piegat A., Pejas J., El Fray I., & Kacprzyk J. (Eds.), ´ Hard and Soft Computing for Artificial Intelligence, Multimedia and Security (1st ed., pp. 216–227). Springer International Publishing 10.1007/978-3-319-48429-7_20

[b41] McConkie G. W., & Rayner K. (1975). The Span of the Effective Stimulus During a Fixation in Reading. Perception & Psychophysics, 17(6), 578–586. 10.3758/BF03203972 0031-5117

[b42] Mills M., Hollingworth A., Van der Stigchel S., Hoffman L., & Dodd M. D. (2011). Examining the influence of task set on eye movements and fixations. Journal of Vision (Charlottesville, Va.), 11(8), 17. 10.1167/11.8.17 1534-7362 21799023 PMC3163592

[b43] Nyström M., & Holmqvist K. (2010). An adaptive algorithm for fixation, saccade, and glissade detection in eyetracking data. Behaviour Research Methods, 42(1), 188–204. Retrieved from http://dx.doi.org/10.3758/BRM.42.1.188 doi:20160299

[b44] Pannasch S., Helmert J. R., Roth K., Herbold A.-K., & Walter H. (2008). Visual Fixation Durations and Saccade Amplitudes: Shifting Relationship in a Variety of Conditions. Journal of Eye Movement Research, 2(2), 1–19.1995-8692

[b45] Peysakhovich V. (2016). Study of pupil diameter and eye movements to enhance flight safety (Unpublished doctoral dissertation). Université de Toulouse, Toulouse, France.

[b46] Qvarfordt P., & Zhai S. (2005). Conversing with the user based on eye-gaze patterns. In Proceedings of the SIGCHI Conference on Human Factors in Computing Systems (pp. 221–230). New York, NY, USA: ACM 10.1145/1054972.1055004

[b47] R Development Core Team (2011). R: A Language and Environment for Statistical Computing [Computer software manual]. Vienna, Austria. Retrieved from http://www.R-project.org/ (ISBN 3-900051-07-0)

[b48] Rayner K., Smith T. J., Malcolm G. L., & Henderson J. M. (2009). Eye Movements and Visual Encoding During Scene Perception. Psychological Science, 20(1), 6–10. Retrieved from http://doi.org/10.1111/j.1467-9280.2008.02243.x doi:PMC2667830 19037907

[b49] Reingold E. M., Charness N., Pomplun M., & Stampe D. M. (2001). Visual span in expert chess players: Evidence from eye movements. Psychological Science, 12(1), 48–55. 10.1111/1467-9280.00309 0956-7976 11294228

[b50] Rosenholtz R., Huang J., Raj A., Balas B. J., & Ilie L. (2012, January). A summary statistic representation in peripheral vision explains visual search. Journal of Vision, 12(4). doi: 10.1167/12.4.14 Rosenholtz, R., Li, Y., & Nakano, L. (2007, August). Measuring visual clutter. Journal of Vision, 7(2), 1–22. doi:10.1167/12.4.14 10.1167/12.4.14 PMC4032502 22523401

[b51] Salthouse T. A., & Ellis C. L. (1980). Determinants of eye-fixation duration. The American Journal of Psychology, 93(2), 207–234. 10.2307/1422228 0002-9556 7406068

[b52] Salvucci D. D., & Goldberg J. H. (2000). Identifying Fixations and Saccades in Eye-tracking Protocols In Proceedings of the 2000 Symposium on Eye Tracking Research & Applications (pp. 71–78). New York, NY: ACM; Retrieved from http://doi.acm.org/10.1145/355017.355028, 10.1145/355017.355028

[b53] Schmitz, C., & LimeSurvey Project Team. (2012). LimeSurvey: An Open Source Survey Tool [Computer software manual]. Hamburg, Germany. Retrieved from http://www.limesurvey.org

[b54] Schnur S., Bektas K., Salahi M., & Çöltekin A. (2010). A Comparison of Measured and Perceived Visual Complexity for Dynamic Web Maps. In Proceedings of the Sixth International Conference on Geographic Information Science (GIScience 2010). Springer International Publishing. Retrieved from https://doi.org/10.5167/uzh-38771 doi:

[b55] Starker I., & Bolt R. A. (1990). A gaze-responsive selfdisclosing display. In Proceedings of the SIGCHI Conference on Human Factors in Computing Systems (pp.3–10). New York, NY, USA: ACM.

[b56] Šterba Z., Šašinka, ˇ C., Stacho ˇ n, Z., Štampach, R., & Morong, K. (2015). Selected Issues of Experimental Testing in Cartography. Brno, Czech Republic: Masaryk University Press. doi: 10.5817/CZ.MUNI.M210-7893-2015

[b57] Trevarthen C. B. (1968). Two mechanisms of vision in primates. Psychologische Forschung, 31(4), 299–337. Retrieved from http://dx.doi.org/10.1007/BF00422717 doi:4973634

[b58] Unema P. J. A., Pannasch S., Joos M., & Velichkovsky B. M. (2005). Time course of information processing during scene perception: The relationship between saccade amplitude and fixation duration. Visual Cognition, 12(3), 473–494. Retrieved from http://dx.doi.org/10.1080/13506280444000409 doi:

[b59] Velichkovsky B. M., Joos M., Helmert J. R., & Pannasch S. (2005). Two Visual Systems and their Eye Movements: Evidence from Static and Dynamic Scene Perception. In CogSci 2005: Proceedings of the XXVII Conference of the Cognitive Science Society (pp. 2283–2288). Stresa, Italy.

[b60] Wehrend S., & Lewis C. (1990). A Problem-oriented Classification of Visualization Techniques In Proceedings of the 1st Conference on Visualization ’90 (pp. 139–143). Los Alamitos, CA: IEEE Computer Society Press; Retrieved from http://dl.acm.org/citation.cfm?id=949531.949553 10.1109/VISUAL.1990.146375

[b61] Weibel R., & Brassel K. E. (2006). Map Generalization—what a difference two decades make In Fisher P. (Ed.), Classics from IJGIS: Twenty years of the International Journal of Geographical Information Science and Systems (pp. 59–65).

[b62] Weiskrantz L. (1972). Behavioural analysis of the monkey’s visual nervous system. Proceedings of the Royal Society of London, 182(1069), 427–455. 10.1098/rspb.1972.0087 0370-1662 4404807

[b63] Wolfe J. M., Alvarez G. A., Rosenholtz R., Kuzmova Y. I., & Sherman A. M. (2011). Visual search for arbitrary objects in real scenes. Attention, Perception & Psychophysics, 73(6), 1650–1671. 10.3758/s13414-011-0153-3 1943-3921 21671156 PMC3153571

[b64] Yarbus A. L. (1967). Eye Movements and Vision. New York, NY: Plenum Press 10.1007/978-1-4899-5379-7

